# A Case of Inverted Uterus

**Published:** 1855-10

**Authors:** Henry Ritchie


### ARTICLE IV.—*A Case of Inverted Uterus.*

WAS called to see Mrs. M., Tuesday evening, the 27th of last August, found a feeble attenuated female, about 30 years of age; upon enquiring as to the nature of her complaint, ascertained that she had been delivered of a living infant, the Saturday evening previous; that after delivery she had repeated fainting fits, with continuous excruciating pain in the lower part of the abdomen, the midwife declaring that she (the patient) was about having another baby, as she had felt and continued to feel its head in the passage.

This history, of course, incited an examination of the vagina, sure enough there was something there filling the passage, and corresponding in size to the head of a fœtus, but the tumor was bleeding, and not quite so hard as a fœtal head; no fontanella could be felt, neither could any os uteri be discovered. The midwife stated that she found this condition after she had taken, or in the act of taking the afterbirth, and then thought it was the patient's stomach coming down, she positively denied having made any traction on the cord.

Upon passing the tumor upwards in the axis of the pelvis, there was a perfect cataract of warm fluid, flowing over the hand, and deluging the bed, evidently the contents of a distended bladder. Handling the tumor, and any degree of pressure upon it called forth piteous exclamations from the poor woman. The pulse of the patient was rapid and feeble, the countenance expressive of great anguish. Examination of the hypogastric region gave no indication of the presence of a post partum uterus, generally so prominent in that region after recent parturition.

A consultation was immediately demanded after unsuccessful efforts to reduce the tumor, intimating at the same time to the friends, the nature of the case, and the woman's critical condition, they requested me to do all that could be done. I therefore went in pursuit of professional aid, after putting a bottle of chloroform in my pocket, determining not to forestal the medical opinion of the gentleman, by any intimation of the nature of the case, merely stating it as an obstetrical case. "What is the matter here?" quoth the Dr. "Matter enough yer honor, the lassie has had a bit of a babe last Saturday, and now there is another one, but it will not come at all at all," replied the old Granny. Then she has had a child, I thought *that* was the difficulty. Will you examine the vagina Dr. An inverted uterus! exclaims the Dr., yes I replied total inversion, this he affirmed with the remark, that there was only one remedy, viz., reduction. Have you chloroform? I have: After fully acquainting her friends with her perilous condition, and giving her an opportunity of being shrived by the priest, we got her fully under the influence of chloroform; but after every manipulation that was thought of had been resorted to, we failed to reduce it. It continued hard, and unyielding; grasping the tumor in the hand, (as far as its bulk would allow), and by this means squeezing the blood out, signally failed also, so that much to our regret, we had to abandon all further efforts at reduction, and only hoped that what we failed to do might be accomplished by some effort of nature.

Wednesday morning, the woman says that she is free from pain, pulse rapid, skin hot, slept some during the night, incontinence of urine, tumor in statu quo, still bleeding, no appetite, ordered wine and beef tea.

Thursday, much the same, excepting skin not so hot, tongue clean, incontinence of urine continues, tumor has ceased to bleed, no appetite, to continue diet as above.

Friday, more feeble, and emaciated, looks wild, says she has no pain, other appearances the same. Sunday morning, is delirious, can hardly be kept in bed, talking incessantly, tender abdomen, hot skin, in fact fever has set in, will not take nourishment, nothing but water.

Monday, is sinking into a typhoid condition, is so violent, as to be almost unmanagable, indifferent attention to her, the nurse and friends afraid of her, my directions so indifferently attended to, that I ceased to visit,—she died the following Monday.

The friends were averse to a *post mortem*, I suppose, for they promised to advise me of her death, with this view—but which I did not ascertain until after her burial.

*Remarks.*—It is much to be regretted that so much antipathy exists against *post mortems*, by the community generally; this case would have been doubly interesting, if the condition of the parts had been declared by actual inspection. I am fully satisfied in my own mind, that it was complete inversion, though my friend, upon hunting up authorities, thinks, that to have been total inversion, the tumor would have projected beyond the vulva. But if it had been only partial, the efforts that we made would no doubt have restored it, and besides in partial inversion, the os uteri ought to have been discovered grasping some part of the inversion. Also upon pressing up the tumor in the vagina, and then by counter pressure upon the abdominal walls, a distinctly defined ring, or indenture about an inch and a half in diameter, could be detected through the abdominal walls.

The tumor in the vagina was slightly smaller at its upper part, but too large even there to be grasped fully by the hand, and did not terminate in a constricted part, but lost itself in the walls of the vagina; the lower part was just within the vulva.

HENRY RITCHIE.

